# Prevalence of syphilis infection in different tiers of female sex workers in China: implications for surveillance and interventions

**DOI:** 10.1186/1471-2334-12-84

**Published:** 2012-04-04

**Authors:** Xiang-Sheng Chen, Qian-Qiu Wang, Yue-Ping Yin, Guo-Jun Liang, Ning Jiang, Li-Gang Yang, Qiao Liu, Yu-Jiao Zhou, Xi-Ping Huan, Wan-Hui Wei, Baoxi Wang

**Affiliations:** 1 National Center for STD Control, Chinese Academy of Medical Sciences and Peking Union Medical College Institute of Dermatology, 12 Jiangwangmiao Street, Nanjing 210042, China; 2 Guangdong Provincial Center for Skin Diseases and STD Control, Guangzhou, China; 3 Hainan Provincial Institute of Dermatology, Haikou, China; 4 Guangxi Center for Diseases Control and Prevention, Nanning, China; 5 Jiangsu Center for Disease Control and Prevention, Nanjing, China

**Keywords:** Syphilis, Prevalence, Typology, Female sex worker, China

## Abstract

**Background:**

Syphilis has made a dramatic resurgence in China during the past two decades and become the third most prevalent notifiable infectious disease in China. Female sex workers (FSWs) have become one of key populations for the epidemic. In order to investigate syphilis infection among different tiers of FSWs, a cross-sectional study was conducted in 8 sites in China.

**Methods:**

Serum specimens (n = 7,118) were collected to test for syphilis and questionnaire interviews were conducted to obtain socio-demographic and behavioral information among FSWs recruited from different types of venues. FSWs were categorized into three tiers (high-, middle- and low-tier FSWs) based on the venues where they solicited clients. Serum specimens were screened with enzyme-linked immunosorbent assay (ELISA) for treponemal antibody followed by confirmation with non-treponemal toluidine red unheated serum test (TRUST) for positive ELISA specimens to determine syphilis infection. A logistic regression model was used to determine factors associated with syphilis infection.

**Results:**

Overall syphilis prevalence was 5.0% (95%CI, 4.5-5.5%). Low-tier FSWs had the highest prevalence (9.7%; 95%CI, 8.3-11.1%), followed by middle-tier (4.3%; 95%CI, 3.6-5.0%, *P *< 0.001) and high-tier FSWs (2.2%; 95%CI, 1.6-2.9%, *P *< 0.001). Factors independently associated with syphilis infection included older age, lower education level, geographic location, lower tier of typology, and injection drug use.

**Conclusions:**

This multi-site survey showed a high prevalence of syphilis infection among FSWs and substantial disparities in syphilis prevalence by the tier of FSWs. The difference in syphilis prevalence is substantial between different tiers of FSWs, with the highest rate among low-tier FSWs. Thus, current surveillance and intervention activities, which have low coverage in low-tier FSWs in China, should be further examined.

## Background

Syphilis has made a dramatic resurgence in China during the past two decades [1]. Heterosexual contacts have become one of primary modes of HIV transmission in China [2,3]. Syphilis has been considered as one of factors to facilitate HIV transmission [4]. Female sex workers (FSWs) are more at risk of having HIV, syphilis and other sexually transmitted infections (STIs). Syphilis has been ranked as the third most prevalent notifiable infectious disease in China. Thus, the syphilis epidemic holds important relevance for infectious disease burden control in China and the region. Assessing the disease burden of syphilis among FSWs is necessary for planning primary and secondary prevention control measures. Syphilis surveillance in China has relied largely on case reports submitted by hospitals or clinics through a website-based case-reporting system [1]. Syphilis prevalence monitoring among specific high-risk populations including FSWs is conducted through HIV sentinel surveillance surveys to provide trends for gauging the effectiveness of prevention efforts [5]. The reported incidence of primary and secondary syphilis (reflecting the intensity of recent transmission) was 11.7 cases per 100, 000 residents in 2009, which had increased by 2.1 times since 2005 [6]. However, case report data may underestimate the true burden of syphilis due to inadequate screening among asymptomatic infected persons and underreporting of the diagnosed cases. Current sentinel surveillance for HIV and syphilis infections among FSWs in China is usually based on recruitment of a convenience sample of FSWs working in entertainment establishments. Based on a list of establishments which are typically used for interventions through outreach work, some venues are selected for recruiting a convenience sample of FSWs for a questionnaire survey and specimen collection. Freelance FSWs are usually not included in the surveillance of most sentinel sites. In this case, the FSWs working at luxurious sex venues (e.g. karaoke bars or hotels) are usually disproportionately recruited. In this paper, we present a multi-site epidemiological study on the prevalence and risk factors of syphilis among FSWs recruited from different typologies of venues in China, and discuss the implications of these findings for surveillance and interventions.

## Methods

### Study sites and survey design

The study was conducted in eight cities in the eastern part (Changzhou and Yangzhou in Jiangsu Province) and southern part (Hezhou and Wuzhou in Guangxi Province, Jiangmen and Maoming in Guangdong Province, and Sanya and Qionghai in Hainan Province) of China. We selected cities to encompass areas where STIs are highly prevalent and where sexual contact is the main route transmission of HIV in China.

The Mega Projects were the China Ministry of Science and Technology (MOST) and Ministry of Health (MOH) projects awarded to pursue the most important public health issues in China. The current study was a baseline survey (a cross-sectional study) for one of the Mega Projects to create cohorts for analyzing the impact of expanded STI care on new HIV infection rate among high-risk groups. FSW settings were mapped and categorized in each study site and a convenience sample design was used to recruit a sample of the FSWs. Each participant was interviewed using a structured questionnaire to collect data on socio-demographic and behavioral information, and then underwent a biologic specimen collection.

### Participants

Between June and September 2009, venues where FSWs solicited clients were mapped and categorized in each study site, and potential participants were identified at the different categories of venues. FSWs were recruited by outreach workers and eligibility requirements included age > 16 years (to encompass FSWs representative of those at risk for STIs); ability to give consent; and having provided commercial sex in sex-work venues or rented apartments for money or goods within the previous three months. FSWs were classified into three subgroups based on the different categories of venues where FSWs solicited clients, i.e. high-tier FSWs (HT-FSWs), middle-tier FSWs (MT-FSWs) and low-tier FSWs (LT-FSWs). High-tier group included FSWs who solicited in karaoke bars, or hotels; middle-tier group included FSWs who solicited in hair salons or barber shops, massage parlors, foot bathing shops, roadside shops, guesthouses, or roadside restaurants; and low-tier group included FSWs who solicited on the street or public outdoor places. MT-FSWs were sampled at a higher rate compared with the HT-FSW, and all accessible and acceptable LT-FSWs were recruited to allow sufficient sample sizes for analysis.

Site staff secured verbal consent from subjects to have blood drawn for free syphilis and HIV testing and to be interviewed with an anonymous and structured questionnaire by the trained outreach workers. All participants tested for syphilis and HIV were informed of their syphilis test results by an outreach team member within a week and informed of their HIV results a few weeks later. Participants with positive tests received counseling messages and were referred to designated clinics for further evaluation and possible treatment according to the national guidelines. No unique identifiers were obtained. A small gift priced at 30 yuan (around 5 US dollars) as an incentive was given to those women who agreed to participate in the survey.

The study protocols were reviewed and approved by the Medical Ethics Committee of the Chinese Academy of Medical Sciences Institute of Dermatology and National Center for Sexually Transmitted Disease Control in Nanjing.

### Laboratory methods

Sera from the participants were evaluated at the STI laboratories of local CDCs or institutes of dermatology and venereology using serologic tests for determining syphilis infection according to the national algorithms. All specimens were first screened for treponemal antibody using an enzyme-linked immunosorbent assay (ELISA). Specimens with positive ELISA underwent a qualitative non-treponemal toluidine red unheated serum test (TRUST) testing, and those specimens with reactive TRUST further underwent a quantitative/titer TRUST testing. For calculating the prevalence of syphilis, the definition of syphilis infection included that specimens must have a positive ELISA and a positive TRUST [7].

Performance of the tests was evaluated on internal quality assurance procedures through re-testing all the serologically positive specimens and 10% of the negative specimens. The tests were found to have a favorable performance in the study sites.

### Statistical analysis

All data from questionnaires, and laboratory results were concurrently double-entered into a computer database using EpiData Software (version 3.0) by independent research assistants, and databases were evaluated for congruency. When database entries conflicted, the original test results and surveys for these cases were retrieved to correct entered data. Prevalence of syphilis infection, with 95% confidence intervals (CIs), was measured. We used univariate and multivariate logistic regression to evaluate factors associated with syphilis infection using backward stepwise elimination. The variables attaining *p *< 0.10 significance in univariate analysis were included in the multivariate regression analysis, retaining only variables achieving *p *< 0.05 significance in the final model. Odds ratios (ORs), with 95% CIs, for risk factors for acquiring the infection were also determined. All statistical analysis was performed using Statistical Program for Social Sciences (SPSS, version 13.0, Chicago, IL) software.

## Results

A total of 7,118 FSWs, including 2,144 (30.1%) HT-FSWs, 3,227 (45.3%) MT-FSWs and 1,747 (24.5%) were enrolled from eight study sites to participate in a baseline survey during June and September 2009. The rates to refuse participating in the study were 8-12% in HT-FSWs, 20-25% in MT-FSWs and 30-35% in LT-FSWs. Overall, 7,118 specimens underwent syphilis ELISA testing followed by TRUST testing for those with positive ELISA. The prevalence of syphilis infection in the study population was 5.0% (95% CI, 4.5%-5.5%).

Table 1 shows syphilis prevalence by selected socio-demographic and behavioral characteristics. In univariate analysis, the prevalence increased with older age and less education (*P *< 0.001 for each). FSWs in Guangxi had a higher prevalence (7.1%; 95%CI, 6.1-8.1%) than those from other 3 provinces. Within Guangxi, Hezhou had the highest prevalence (12.2%; 95%CI. 10.0-14.5%). Those FSWs who were separated, divorced or windowed in marital status had a higher prevalence (9.9%; 95%CI, 5.8-14.0%) than those who were married (7.0%; 95%CI, 6.0-8.0%) or never married (3.5%; 95%CI, 3.0-4.1%). Syphilis prevalence was highest among LT-FSWs (9.7%; 95%CI, 8.3-11.1%), followed by MT-FSWs (4.3%; 95%CI, 3.6-5.0%, *P *< 0.001) and HT-FSWs (2.2%; 95%CI, 1.6-2.9, *P *< 0.001). Syphilis prevalence was also higher among persons with a positive HIV test (14.0%; 95%CI, 3.6-24.3%) than those with a negative HIV test (4.9%; 95%CI, 4.4-5.5; *P *= 0.007). A small number of FSWs reported a history of needle-sharing, which limited our ability to make reliable estimates of syphilis prevalence among FSWs who shared needles. However, the prevalence was higher among FSWs who reported injecting drugs (25.0%; 95%CI, 6.0-44.0%) than those who did not (4.9%; 95%CI, 4.4-5.4%, *P *< 0.001). The difference in syphilis prevalence between FSWs who reported consistent use of a condom and no use of a condom in the past month was not statistically significant (*P *= 0.257).

**Table 1:** Prevalence of syphilis by demographic and behavioral characteristics and HIV infection (univariate analysis)

Characteristics	Number*	Prevalence % (95%CI)	*P *value	OR (95%CI)
Total	7118	5.0 (4.5-5.5)		
Age in years (median, interquartile range)	24 (16-56)		< 0.001	1.08 (1.07, 1.10)
Age group			< 0.001	
16-19	1015	1.7 (1.0, 2.5)		1.0 (Reference)
20-29	4197	4.0 (3.0, 4.6)		2.42 (1.46, 4.00)
30-39	1519	7.8 (6.0, 9.2)		4.98 (2.97, 8.33)
40-	387	14.4 (11.0, 18.0)		9.85 (5.63, 17.23)
Province			< 0.001	
Jiangsu	1791	3.6 (2.8-4.5)		1.0 (Reference)
Guangdong	1361	4.0 (2.9-5.0)		1.10 (0.76, 1.59)
Guangxi	2515	7.1 (6.1-8.1)		2.03 (1.52, 2.72)
Hainan	1451	3.9 (2.9-4.9)		1.10 (0.77, 1.59)
Site city			< 0.001	
Yangzhou	929	0.8 (0.2-1.3)		1.0 (Reference)
Changzhou	862	6.7 (5.1-8.4)		9.49 (4.31, 20.92)
Jiangmen	533	3.0 (1.6-4.6)		4.07 (1.66, 9.95)
Maoming	828	4.6 (3.2-6.0)		6.39 (2.84, 14.38)
Wuzhou	1706	4.7 (3.7-5.7)		6.49 (2.99, 14.11)
Hezhou	809	12.2 (10.0-14.5)		18.33 (8.46, 39.69)
Qionghai	643	4.4 (2.8-5.9)		6.25 (2.71, 14.40)
Sanya	808	3.6 (2.3-4.9)		4.89 (2.13, 11.23)
Education			< 0.001	
Primary or less	1140	9.4 (7.7-11.1)		4.42 (2.91, 6.72)
Junior high school	4727	4.6 (4.0-5.2)		2.04 (1.38, 3.02)
Senior high school and above	1243	2.3 (1.5-3.2)		1.0 (Reference)
Marital status			< 0.001	
Never married	4262	3.5 (3.0-4.1)		1.0 (Reference)
Married	2641	7.0 (6.0-8.0)		2.08 (1.67, 2.59)
Separated/divorced/widowed	202	9.9 (5.8-14.0)		3.03 (1.86,4.95)
Ethnicity			0.452	
Han	6263	5.0 (4.5-5.5)		1.0 (Reference)
Minority	827	5.6 (4.0-7.2)		1.13(0.82, 1.55)
Household registration(hukou)			0.322	
Local	1859	4.6 (3.7-5.6)		1.0 (Reference)
Not local	5246	5.2 (4.6-5.8)		1.13(0.88, 1.46)
Typology			< 0.001	
High-tier	2144	2.2 (1.6-2.9)		1.0 (Reference)
Middle-tier	3227	4.3 (3.6-5.0)		1.95 (1.40, 2.73)
Low-tier	1747	9.7 (8.3-11.1)		4.76 (3.43, 6.60)
Having had any STI symptoms past year			0.658	
Yes	2892	4.9 (4.1-5.7)		0.95 (0.77, 1.18)
No	4200	5.1 (4.4-5.8)		1.0 (Reference)
Consistent condom use in past month			0.254	
Yes	3282	5.4 (4.7-6.2)		1.14 (0.91,1.42)
No	3266	4.8 (4.0-5.5)		1.0 (Reference)
Injecting drug use			< 0.001	
Yes	20	25.0 (6.0-44.0)		6.39 (2.31, 17.69)
No	7063	4.9 (4.4-5.4)		1.0 (Reference)
HIV infection			0.007	
Positive	43	14.0 (3.6-24.3)		3.11 (1.30, 7.41)
Negative	7059	4.9 (4.4-5.5)		1.0 (Reference)

*The sum of numbers in some subgroups was less than 7118 because some participants did not respond to some questions.

*CI *confidence interval, *OR *odds ratio.

In multivariate logistic regression analysis (Table 2), the factors associated with syphilis prevalence were older age (AOR > 2.62 as compared with the age group of 16-19 years, *P *< 0.001), study site rather than Yangzhou site (AOR > 4.17 as compared with the Yangzhou site, *P *< 0.001), lower education level (AOR > 1.56 as compared with senior high school or above, *P *< 0.03), lower tier in FSW typology (AOR > 1.68 as compared with high-tier FSWs, *P *< 0.008), and injection drug use (AOR = 6.17 as compared with no injection drug use, *P *= 0.001). Multivariate logistic regression analysis showed that the LT-FSWs tended to be older with age of 40 years or above (AOR = 1.81 as compared with age of 16-19 years, *P *< 0.001), less education level (AOR > 2.54 as compared with senior high school or above, *P *< 0.001), married or separated/divorced/widowed status (AOR = 1.86 as compared with non-married status, *P *< 0.001), non-Han minority (AOR = 1.29 as compared with Han minority, *P *= 0.005), and local residence (AOR = 2.11 as compared with non-local residence, *P *< 0.001), Table 3.

**Table 2:** Multivariate analysis of factors associated with syphilis infection

Factor	Adjusted OR (95%CI)	P
Age group in years		
16-19	1.0 (Reference)	-
20-29	2.62 (1.57, 4.36)	< 0.001
30-39	4.37 (2.57, 7.42)	< 0.001
40-	7.81 (4.36, 14.01)	< 0.001
Site city		
Yangzhou	1.0 (Reference)	-
Changzhou	14.35 (6.43, 32.03)	< 0.001
Jiangmen	9.51 (3.76, 24.08)	< 0.001
Maoming	10.62 (4.61, 24.47)	< 0.001
Wuzhou	4.17 (1.90, 9.17)	< 0.001
Hezhou	13.54 (6.17, 29.71)	< 0.001
Qionghai	9.44 (4.00, 22.29)	< 0.001
Sanya	8.14 (3.48, 19.02)	< 0.001
Education		
Primary or less	2.10 (1.34, 3.30)	0.001
Junior high school	1.56 (1.04, 2.35)	0.031
Senior high school and above	1.0 (Reference)	-
Typology		
High-tier	1.0 (Reference)	-
Middle-tier	1.68 (1.15, 2.46)	0.008
Low-tier	4.05 (2.60, 6.31)	< 0.001
Injecting drug use		
Yes	6.17 (2.13, 17.81)	0.001
No	1.0 (Reference)	-

*OR *odds ratio, *CI *confidence interval.

**Table 3:** Multivariate analysis of factors associated with low FSW typology

Factor	Low vs. middle/high tiers AOR, 95%CI	*P *value
Age group in years		
16-19	1.00 (Reference)	--
20-29	0.71 (0.59, 0.86)	< 0.001
30-39	1.26 (0.99, 1.61)	0.06
40-	1.81 (1.33, 2.46)	< 0.001
Education		
Primary or less	6.45 (5.10, 8.15)	< 0.001
Junior high school	2.54 (2.06, 3.13)	< 0.001
Senior high school and above	1.0 (Reference)	--
Marital status		
Never married	1.0 (Reference)	--
Married	1.86 (1.58, 2.18)	< 0.001
Separated/divorced/widowed	2.15 (1.54, 3.01)	< 0.001
Ethnicity		
Han	1.0 (Reference)	--
Minority	1.29 (1.08, 1.55)	0.005
Household registration (*hukou*)		
Local resident	2.11 (1.86, 2.40)	< 0.001
Not local resident	1.0 (Reference)	--

*AOR *adjusted odds ratio, *CI *confidence interval.

## Discussion

Commercial sex is illegal in China. By 2005, the Chinese Center for Disease Control and Prevention (CDC) estimated that there were 2.8 to 4.5 million FSWs nationwide [8]. Risk behaviors and socio-demographic characteristics of FSWs linked to the sex trade may be associated with risk of STI infection, health care seeking behaviors, and/or social stigma [9,10]. There have been several ways to classify women who provide sex for money or gifts. A study has categorized FSWs into seven typologies according to their workplace and organization, income and demographics, and approximate sexual risk [11], while other studies have categorized them into 3 tiers simply based on sex work venues and socio-economic contexts [12,13]. Despite the fact that no consensus on FSW typologies has been met in China, typology of FSWs is important for design of surveillance and intervention programs.

A substantial prevalence of syphilis was found among FSWs in the study areas. These findings are consistent with the results reported elsewhere among FSWs with 5.3% in Sichuan [14], and 3% in Shandong [15], but are much higher than the median level of national prevalence (0.5-1.8%) [5]. The prevalence varies significantly by different tier of FSWs, showing a higher prevalence among LT-FSWs (9.7%) than MT-FSWs (4.3%) or HT-FSWs (2.2%). A systematic review of published studies found that low-tier FSWs are approximately twice as likely to have syphilis [16]. Some studies have found extremely high syphilis prevalence in this group, ranging from 10-38% [13,17]. Studies have shown that the FSWs working in low-tier venues tended to have greater number of clients, but infrequently used condoms due to extra payment for unsafe sex [17-19]. Low-tier FSWs either independently solicit clients on streets (street-walking FSWs) or find clients (some knew each other) through nearby construction sites and factories [11]. Our results further indicate that older women are more likely to be infected with syphilis, and the women with less education are at a greater risk of infection. A 2006 national probability survey among men 15-49 years old indicated that the prevalence of visiting FSWs in the previous year was 4.2% overall, with 7.2% in urban areas and 1.8% in rural areas [20]. Although we do not have data on the percentages of Chinese men who visit each of the different tiers of FSWs, it has been found from observations in several areas that the clients of low-tier FSWs are usually migrants and elder men. It is known from our study that there are significant differences in terms of socio-demographic characteristics between different tiers of FSWs, indicating that low-tier FSWs tend to be older women with low education levels, and the women from local areas and non-Han minorities. These differences are important determinants for designing surveillance and intervention programs. The low-tier subgroups of FSWs are less well represented in current HIV and STI surveillance programs and are also less likely to be covered by current intervention programs in China. The significant differences in terms of demographic characteristics and prevalence of syphilis between FSWs recruited from low-tier sex work venues and those from middle or high-tier venues warrants consideration of what kind of population we should recruit and/or where we should recruit them to represent the FSWs for surveillance purposes. However, the interpretation of epidemiological and behavioral data emerging from such settings is challenging due to significant selection bias. The high prevalence of the syphilis infections in the study populations further emphases the importance of effective curative and preventive STI services for the FSW population. Syphilis surveillance and monitoring of the response to the STI epidemic may be improved by carefully considering and selecting appropriate target populations to represent women at high risk.

The strengths of this study include (1) the inclusion and classification of different tiers of FSWs in the study; (2) the utilization of community-based or outreach-based recruitment strategies; and (3) the multi-site survey consisting of 8 middle-size cities in China. There are also limitations to be addressed. First, although the study was carried out in eight cities, the socio-economic and behavioral characteristics of FSWs in the study areas may be different from other Chinese cities. Any generalization of the results from this study should therefore be made with caution. Second, the study was not able to use a random sampling method to recruit the FSWs, likely resulting in the sample bias. Third, high rates of FSWs to refuse participating in the study (i.e., 8-12%, 20-25% and 30-40% among HT-, MT- and LT-FSWs, respectively) may result in potential selection bias. Fourth, the sample size for this study may not be large enough for the subgroup analysis to show potential associations between the infections and some risk factors. Fifth, this study included only limited information on risk behaviors, and data regarding sexual behaviors and condom use is subject to self-reporting bias and social response bias due to the sensitivity of these topics. Finally, this study did not ascertain an association between syphilis and HIV infection because the number of FSWs with HIV infection was small, although some studies in China have shown that HIV infection is independently associated with syphilis infection [20]. In addition, some potential factors related to the infection, such as condom use with regular non-client sexual partners and the number of clients per week, were not included in the study.

As FSWs are an important source of infection and probably the bridge population for the heterosexual transmission of syphilis and HIV, the findings of this study have a number of important implications. First, this study shows a substantial prevalence of syphilis infection among FSWs and much higher prevalence among LT- and MT-FSWs. The patterns of infection suggest the importance of prioritizing the MT- and LT-FSWs in the current program for interventions (health education, condom promotion and STI care services) in FSWs. Second, this study suggests a re-appraisal of the current HIV/STI surveillance system in terms of sampling of women at high risk in China to enable more representative, valid and reliable prevalence estimates to be generated. Third, this study highlights our limited knowledge of the epidemiology of syphilis and likely other STIs among LT-FSWs in China. LT-FSWs are thought to be a potential and key co-factor for sustaining the spread of STIs in high-risk populations and for bridging the epidemic from high-risk groups to the general population. However, they are usually neglected by routine intervention programs. This underscores the urgency of including this neglected population in preventive interventions for reducing STI infection. The current HIV/STI intervention programs for female sex workers are urgently needed to improve for increasing access to MT- and LT-FSWs in China through providing a comprehensive intervention package. The package should include not only health education materials and condoms but also STI and reproductive health care including syphilis testing and counseling; and delivery of the package should be conducted by not only the solitary CDC outreach teams but also medical personnel and non-governmental organizations.

## Conclusions

A substantial prevalence of syphilis in FSWs, particularly in low-tier FSWs, is a serious public health concern. Syphilis control in this population through universal screening of syphilis and integrating syphilis testing into VCT services, STI risk reduction education, and aggressive condom promotion programs are urgently needed. Much can and should be done to stem the syphilis epidemic and potential uptake of the co-epidemic of syphilis and HIV in FSWs. The Chinese Ministry of Health has already issued a national 10-year plan for syphilis control and prevention, where strengthening the surveillance system in high risk groups as well as comprehensive interventions among FSWs are priorities in order to curb the epidemic in China.

## Competing interests

The authors declare that they have no competing interests.

## Authors' contributions

XSC, YPY, QQW, GJL, NJ and BW and conceived the study and participated in study design. LGY, QL, YJZ and XPH participated in data collection and interpretation. WHW coordinated the laboratory testing of syphilis infection and helped to interpret the data. XSC and NJ performed the statistical analysis. XSC and YPY prepared the manuscript, and NJ, QQW and BW helped to draft the manuscript. All authors read and approved the final manuscript.

## Pre-publication history

The pre-publication history for this paper can be accessed here:

http://www.biomedcentral.com/1471-2334/12/84/prepub

## References

[B1] Chen ZQ Zhang GC Gong XD Lin C Gao X Liang GJ Yue XL Chen XS Cohen MS Syphilis in China: results of a national surveillance programme Lancet 2007 369 132 138 10.1016/S0140-6736(07)60074-9 17223476 PMC7138057

[B2] Yang H Li X Stanton B Liu H Liu H Wang N Fang X Lin D Chen X Heterosexual transmission of HIV in China: a systematic review of behavioral studies in the past two decades Sex Transm Dis 2005 32 270 280 10.1097/01.olq.0000162360.11910.5a 15849527 PMC1791011

[B3] Wang N Wang L Wu Z Guo W Sun X Poundstone K Wang Y Estimating the number of people living with HIV/AIDS in China: 2003-09 Int J Epidemiol 2010 39 Suppl 2 ii21 ii28 21113033 10.1093/ije/dyq209 PMC2992614

[B4] Powers KA Poole C Pettifor AE Cohen MS Rethinking the heterosexual infectivity of HIV-1: a systematic review and meta-analysis Lancet Infect Dis 2008 8 553 563 10.1016/S1473-3099(08)70156-7 18684670 PMC2744983

[B5] Wang L Ding ZW Ding GW Guo W Wang L Qin QQ Li DM Wang LY Yan RX Hei FX Data analysis of national HIV comprehensive surveillance sites among female sex workers from 2004 to 2008 Zhonghua Yu Fang Yi Xue Za Zhi 2009 43 1009 1015 20137527

[B6] Chen XS Peeling RW Yin YP The epidemic of sexually transmitted infections in China: Implications for control and future perspectives BMC Med 2011 9 111 10.1186/1741-7015-9-111 21975019 PMC3203037

[B7] Yin YP Guidelines for Laboratory Diagnosis of Sexually Transmitted Diseases 2007 Shanghai: Shanghai Science and Technology Publishing House

[B8] Lu F Wang N Wu Z Sun X Rehnstrom J Poundstone K Yu W Pisani E Estimating the number of people at risk for and living with HIV in China in 2005: methods and results Sex Transm Infect 2006 82 Suppl 3 iii87 iii91 16735299 10.1136/sti.2006.020404 PMC2576728

[B9] Choi KH Zheng X Zhou H Treatment delay and reliance on private physicians among patients with sexually transmitted diseases in China Int J STD AIDS 1999 10 309 315 10.1258/0956462991914177 10361920

[B10] Lin SC Ma YG Liao Q A study on relationship between first visitingdoctor behaviors of socio-demographic characteristics of STD patients Chin J STD AIDS Prev Control 2002 8 162 163

[B11] Huang Y Henderson GE Pan S Cohen MS HIV/AIDS risk among brothel-based female sex workers in China: assessing the terms, content, and knowledge of sex work Sex Transm Dis 2004 31 695 700 10.1097/01.olq.0000143107.06988.ea 15502679

[B12] Zhou ZG Wang H Wang B Zhu BY Gan Q A survey of behavioral characteristics and prevalence of STIs among 480 female sex workers in Guangxi Chin J Health Edu 2010 26 522 524

[B13] Yang P Wang QQ Peng H A survey of syphilis and HIV infection in medium-low income female sex workers China J Lepr Skin Dis 2009 25 174 176

[B14] Yang C Latkin C Luan R Wang C Nelson K HIV, syphilis, hepatitis C and risk behaviors among commercial sex male clients in Sichuan province, China Sex Transm Infect 2010 86 559 564 10.1136/sti.2009.041731 20826867 PMC4487621

[B15] Liao M Jiang Z Zhang X Kang D Bi Z Liu X Fu J Zhang N Mao W Jiang B Jia YJ Syphilis and methamphetamine use among female sex workers in Shandong Province, China Sex Transm Dis 2011 38 57 62 10.1097/OLQ.0b013e3181ebb475 20679964

[B16] Chang H Zhi X Chen X-S Cohen MS Systematic review and meta-analysis of syphilis seroprevalence among female sex workers in China 2010 Bethesda: NIH Fogarty International Clinical Scholar Conference

[B17] Wang Q Yang P Gong XD Jiang J Yang B Syphilis prevalence and high risk behaviors among female sex workers in different settings China J AIDS STDs 2009 15 398 400

[B18] Yang X Xia G Gender, work, and HIV risk: determinants of risky sexual behavior among female entertainment workers in China AIDS Educ Prev 2006 18 333 347 10.1521/aeap.2006.18.4.333 16961450

[B19] Yang X Xia G Li X Latkin C Celentano D Social influence and individual risk factors of HIV unsafe sex among female entertainment workers in China AIDS Educ Prev 2010 22 69 86 10.1521/aeap.2010.22.1.69 20166789 PMC2826716

[B20] Lu F Jia Y Sun X Prevalence of HIV infection and predictors for syphilis infection among female sex workers in southern China Southeast Asian J Trop Med Public Health 2009 40 263 272 19323011 PMC2745968

